# Automatic classification of fungal-fungal interactions using deep leaning models

**DOI:** 10.1016/j.csbj.2024.11.027

**Published:** 2024-11-14

**Authors:** Marjan Mansourvar, Jonathan Funk, Søren Dalsgård Petersen, Sajad Tavakoli, Jakob Blæsbjerg Hoof, David Llorente Corcoles, Sabrina M. Pittroff, Lars Jelsbak, Niels Bjerg Jensen, Ling Ding, Rasmus John Normand Frandsen

**Affiliations:** Department of Biotechnology and Biomedicine (DTU Bioengineering) Technical University of Denmark, Søltofts Plads, Kgs. Lyngby 2800, Denmark

**Keywords:** Fungal growth, Deep learning, Machine learning, Computer vision, Automation, Biocontrol

## Abstract

Fungi provide valuable solutions for diverse biotechnological applications, such as enzymes in the food industry, bioactive metabolites for healthcare, and biocontrol organisms in agriculture. Current workflows for identifying new biocontrol fungi often rely on subjective visual observations of strains’ performance in microbe-microbe interaction studies, making the process time-consuming and difficult to reproduce. To overcome these challenges, we developed an AI-automated image classification approach using machine learning algorithm based on deep neural network. Our method focuses on analyzing standardized images of 96-well microtiter plates with solid medium for fungal-fungal challenge experiments. We used our model to categorize the outcome of interactions between the plant pathogen *Fusarium graminearum* and individual isolates from a collection of 38,400 fungal strains. The authors trained multiple deep learning architectures and evaluated their performance. The results strongly support our approach, achieving a peak accuracy of 95.0 % with the DenseNet121 model and a maximum macro-averaged F1-Score of 93.1 across five folds. To the best of our knowledge, this paper introduces the first automated method for classifying fungal–fungal interactions using deep learning, which can easily be adapted for other fungal species.

## Graphical Abstract

**Figure:**
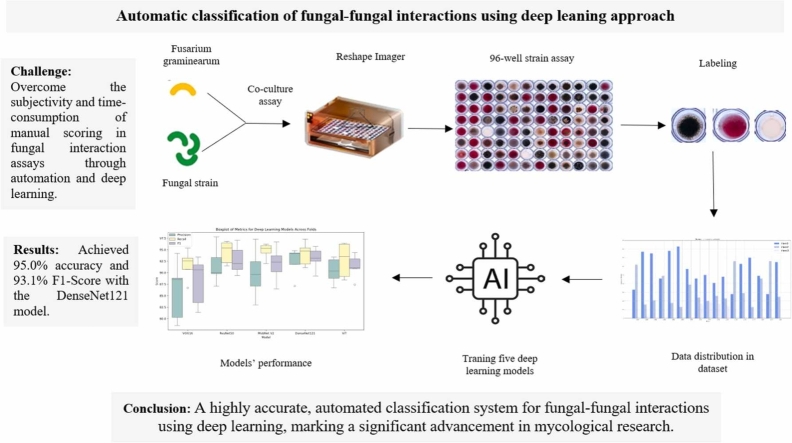

## 1 Introduction

Since the discovery of penicillin, the kingdom of fungi has served as an invaluable source for novel bioactive metabolites and enzymes [1]. These biological treasures have been harnessed for a wide array of applications across various industries, including the production of enzymes and commodity chemicals for industrial and food biotechnology, as well as drugs for pharmaceutical biotechnology[2]. Furthermore, fungi and their derived biologicals find application as biostimulants and biological pesticides in agriculture [3].

The utilization of biological control organisms to manage crop diseases caused by fungi, bacteria, and animal pests has gained increasing attention over the last decade. This development has primarily been driven by a desire to reduce the use of synthetic pesticides, increase the efficiency and reduce the cost of disease management [4], [5]*.* In 2022, the European Commission adopted the Green Deal proposal, aiming to reduce synthetic pesticide use by 50 % by 2030[6]. This has created additional incentives to identify efficient biological solutions for disease management, particularly in critical areas such as row crops (e.g., cereals) [7].

Two major fungal pathogens in cereals are *Fusarium* spp. and *Zymoseptoria* spp., both causing significant losses in harvest yields and reducing the quality of the harvested crop [8]. Members of the *Fusarium* species complex are known to cause *Fusarium* head blight in wheat, barley and oats, *Fusarium* crown rot in wheat and barley and *Fusarium* wilt in a broad array of cereals. In addition to the direct yield losses caused by misgrowth, infections by *Fusarium* sp. are most often associated with the accumulation of regulated mycotoxins, such as deoxynivalenol, NIV, and zeralenone, in the harvested crops[9]. *Zymoseptoria tritici* (formerly *Mycosphaerella graminicola)* is the primary cause of ‘Septoria tritici blotch’, also known as a leaf spot disease, which is the most significant disease affecting wheat production [10]. Control of these fungal pathogens relies on integrated pest management approaches, which combine crop rotation, cultivation practices, use of resilient crop cultivars and targeted applications of synthetic pesticides.

Removing synthetic pesticides without introducing biological alternatives is expected to increase the incidence of disease, resulting in reduced harvest yields, estimated to 30 % for wheat in Europe [11]. The lowered productivity per hectare will increase the needed land to produce the same quantity of crops, resulting in increased resource consumption and a larger negative environmental footprint of food production. Finding biological control means, either whole cell-based, metabolites or enzyme-based, is hence imperative to secure food supply, ensure the economy for the producers, and avoid increasing the footprint of food production.

Filamentous fungi play a key role in many ecological niches, including field soils and crop plants, where fungi compete for resources [12]. In several cases, fungal secondary metabolites (SMs) have been shown to serve as important factors in pathogen-fungal interactions, either by serving as antagonist agents or signals or signaling-disrupting molecules [13]. Certain fungal secondary metabolites exhibit potent antagonistic properties against microbial plant pathogens, making them highly appealing for crop protection applications [14].

The search for new biological control organisms typically relies on the screening for organisms that can inhibit the in vitro growth of the pathogen [15], warranting the availability of an easy-to-score interaction assay and a source of relevant microorganisms. The IBT strain collection (Technical University of Denmark, World Federation of Culture Collections no. 758) contains 38,000 isolates covering approximately 1800 species[16], where the individual strain is supported by a generous meta-data package, including polyphasic identification, data on collection site and dates, growth profiles on various media, and for metabolomics data (either LC-DAD, or LC-DAD-MS2). The IBT collection has not previously been subjected to functional screening for antifungal compounds and hence constitutes an unexplored source of biocontrol organisms.

Current approaches for evaluating the outcome of pathogen-fungal interaction assays typically rely on manual inspection and scoring by humans. This process is not only time-consuming but also introduces subjectivity, significantly reducing reproducibility within and across laboratories. Given the size of the IBT collection and our desire to test a significant portion of individual strains for their ability to control fungal pathogens, we focused on developing an automated classification tool to fungal-pathogen interactions, specifically targeting *Fusarium graminearum*.

Computer vision and image processing has been used in the biology field since the 1960s, initially for the automatic detection and monitoring of cells undergoing mitosis in visual classification tasks [17]. However, the field has witnessed remarkable progress in recent years, primarily driven by advancements in machine learning approaches [18]. As a result, computer vision techniques have found broader utilization across various biological applications. Notably, in cell biology research, computer vision algorithms and machine learning techniques have been extensively employed in the field of drug discovery. High-content screening, for instance, involves the automated detection and individual analysis of cells from fluorescent microscopy images using computer algorithms and machine learning techniques [19]. Similar approaches have been applied for the automated enumeration of bacteriophages in petri dish assays and for the analysis of colorimetric immunoassays [20]. Additionally, image processing methods have been used for the detection of fungal contamination in agricultural products, as demonstrated by Ziyaee et al., who compared different techniques for segregating peanut seeds infected by aflatoxin-producing fungi [21]. Another potential area of application for automated phenotypical analysis by computer vision would be the scoring of pathogen-fungal interaction based on image analysis. The combination of computer vision and machine learning, coupled with the advancement of enhanced algorithms, is poised to emerge as a focal point for prospective research within the domain of the mycology [22], [23]. Conventional neural network approaches in the classification and identification of fungi employ a region selection strategy based on sliding windows [24]. However, this method lacks specificity and is characterized by high complexity. Furthermore, the manually designed feature extractor exhibits limited robustness in accommodating the diverse nature of targets. The heightened computational capabilities of contemporary computers, notably Graphics Processing Units (GPUs), have rekindled interest in deep learning methodologies [25]. This resurgence is particularly evident in the successful application of convolutional neural networks within the realm of computer vision. Presently, object detection tasks predominantly leverage convolutional neural network-based methods, offering the advantage of obviating the need for manual feature design during image feature extraction [26]. Deep Neural Networks (DNN) have emerged as a powerful approach for classification and clustering tasks in big data analysis [27]. Within deep learning, convolutional neural networks (CNNs) have been particularly successful in analysing visual images. CNNs are a type of deep neural network that excel at learning high-level features through convolutions. One of the main advantages of CNNs is their ability to develop internal representations of two-dimensional signals, enabling them to capture position and scale variant structures in data, which is crucial for image analysis. CNNs have gained widespread popularity due to their exceptional performance in tasks such as image recognition and analysis [28].

In comparison to traditional machine learning methods, CNNs have shown significant improvements in classification accuracy [29]. CNN based image analysis can potentially improve the accuracy and efficiency of classification pathogen-fungal interaction assays, compared to traditional methods. The utilization of convolutional neural networks in the classification and identification of fungal species has been a concept under consideration for an extended period [23]. However, the recent development, and successes, with the use of machine learning approaches for image analysis have to the best of our knowledge not been used to study microbial-microbial interaction.

In this study we developed an automatic AI computer vision system to analyze images of co-cultures and automate classification of the outcome of such interactions. The method aims to address the challenge of classification of large pathogen-fungal interaction datasets. Our approach involves using a fully automated method for classification of images from a high-throughput pathogen-fungal competition assays based on the deep learning approaches. Specifically, we focus on classification the fungal candidate strain's ability to suppress growth of the plant pathogen *Fusarium graminearum*.

## 2 Materials and methods

### 2.1 Fungal strains assay and manual classification

The fungal strains used in this study were obtained from the IBT (Institute for Biotechnology) strain collection at the Technical University of Denmark (DTU), which encompasses a collection of about 38,400 fungal species[30]. The competition assay involved the co-cultivation of fungal candidates from the IBT collection with the plant pathogen *Fusarium graminearum* on solid medium in 96-well plates. The strains were co-cultivated on Potato Dextrose Agar (PDA) at a temperature of 25 degrees Celsius for a period of 6 days prior to image capture. The individual well (Ø = 5.5 mm) contained 0.15 mL PDA medium. *Fusarium graminearum* was inoculated at a density of 2000 spores per well, while the candidate strain was introduced at a similar density by placing 0.4 μL droplets on the PDA surface, 0.5 mm apart, in four spots. As control, the individual candidate fungal strain and the *Fusarium graminearum* pathogen were in parallel cultivated separately using similar conditions.

To minimize potential cross-contamination and reduce the influence of volatile compounds among wells, each plate was sealed with a lid immediately after inoculation, and images were captured with the lid in place. Both the plate and lid typically developed condensation rings, creating a natural barrier to restrict volatiles within each well. Although no specific assessment of empty wells was conducted in this study, we believe that the seal and lid adequately contained volatiles. Future experiments could benefit from including empty control wells to monitor potential cross-contamination effects and further ensure isolation.

The fungal growth in the individual wells of the 96-well plates was assessed by visually analyzing the color and mycelium morphology of the two co-cultured fungi. A three-state classification regime were applied to identify the winner of the interaction: If the color and morphology in a well closely resembled that of the candidate fungus cultured alone, the interaction was classified as a win for the candidate strain. On the other hand, if the morphology and color more closely resembled that of the pathogen, it was classified as a loss for the candidate. In cases where the morphology and color resembled a mix of the two strains, or no growth was observed it was classified as a draw.

### 2.2 Image acquisition

**Fig. 1:**
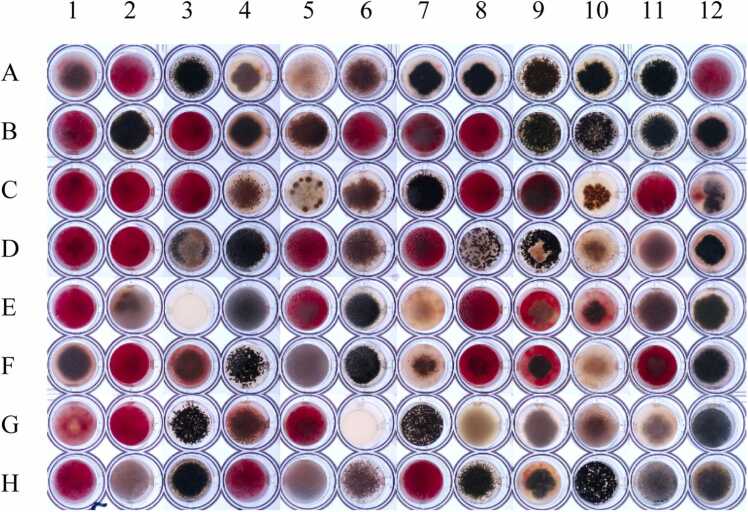
Image of a 96-well plate with the competition assays following 6 days of incubation. All wells were inoculated with *F. graminearum* spores (resulting in red mycelium) and 96 different candidate fungi from the IBT collection. The image (output from Reshape platform) is a composite of 24 individual images each covering 2 × 2 wells. The stitched images was divided into 96 individual images, each covering one sample well.

The images for the analysis were acquired using an Imaging robot (Reshape Biotech) that include a 12 megapixels camera mounted on a computer control rail system with XY movement, and a versatile elimination setup, including Bottom Light and Top Light functionalities. Images were recorded using the Top Light setting, enabling the observation and differentiation of colors to distinguish empty wells, pathogens, and candidates. The light intensity was set to medium during the data collection period. The robot recorded images of four wells simultaneously and then moved the camera to the next location on the plate, at 12-hour intervals over a span of 6 days (Fig. 1). The resulting 24 JPEG/RAW images per assay plate were stitched together using Reshape Biotech's image package. The resulting image of the 96-well plate (Fig. 1) was further processed by splitting it into individual JPEG/RAW images of the 96 individual wells, utilizing a custom Python script that calls the OpenCV library [31].

### 2.3 The dataset

**Fig. 2:**
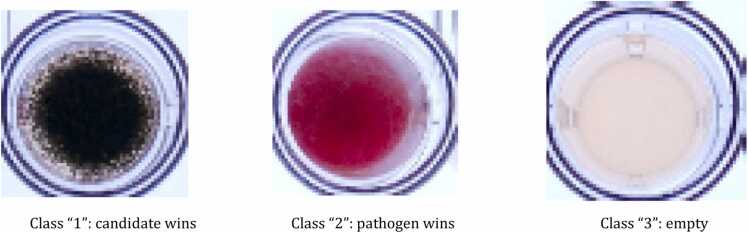
Examples of the dataset of images and their classes.

A total of 23,000 single well images were captured following the procedure described in the previous section. Out of these, 1564 images were manually annotated by biological experts from 17 distinct 96-well plates. They assigned one of three labels to indicate the result of the competition assay: 'class 1′ (representing candidate wins), 937 images labeled as '2' (representing pathogen wins), and 71 images labeled as '3' (representing empty wells). Examples of these annotated images can be seen in Fig. 2.

**Fig. 3:**
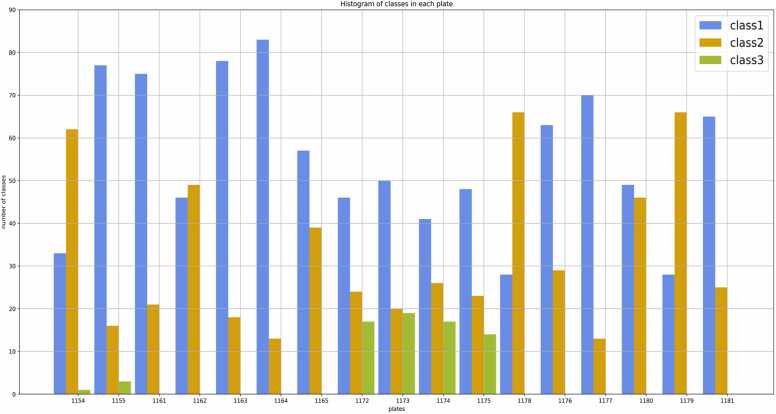
Data Distribution of three distinct classes within the dataset. The dataset's imbalance is evident, particularly concerning class 3. This imbalance poses a challenge during the training of models. X axis indicates the 96 well plate number and Y axis shows the distribution of each class on each plate.

Fig. 3 visually represents the distribution of three distinct classes within the dataset for all plates. The image dataset exhibits a noticeable imbalance, a well-recognized issue known to potentially adversely affect classification algorithms [26]. By taking a close look at the histogram of classes in each plate, we can observe that some plates do not have any wells from class 3. To address this concern, we utilized a data augmentation method [27]. Specifically, we expanded the dataset by applying rotation, vertical and horizontal flips, as well as a combination of both. Augmenting the image dataset not only mitigated imbalanced data but also addressed the overfitting phenomenon [32].

### 2.4 Model Training

#### 2.4.1 Image Preprocessing

**Fig. 4:**
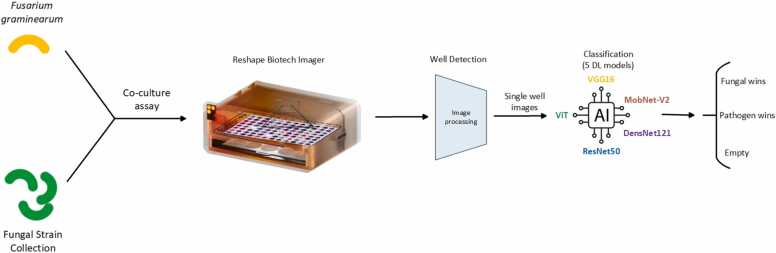
The prediction workflow for classification of fungal images. The DNN model architectures include five well-known deep learning algorithms: ViT, VGG16, ResNet50, DenseNet121, and MobileNet-V2.

All images were initially processed using an input sequencer to identify any outlier images. To ensure uniformity, image augmentation techniques were utilized, resizing the images to dimensions of 224 × 224 × 3 [33]. The number of points generated depends on the resolution of the input image. The dataset was manually curated, and images with resolutions different from 224 × 224 pixels were adjusted to the desired dimensions using the 'resizer function' in the OpenCV Python package to align with the model architecture. Following image standardisation further processing was conducted as outlined in Fig. 4.

Color was an important feature in classifying outcomes, particularly for class 2, and a consistent color assessment procedure was followed during annotation to ensure standardization. Comparisons were made with controls to evaluate additional features beyond color, such as mycelial growth, spore formation, and pigment production, which aided in distinguishing between classes. Based on expert experience in annotation, other indicators were also assessed, such as growth inhibition (absence of growth) and whether the candidate strain grew over the pathogen within the well. This additional evaluation helped ensure a robust and accurate classification by combining visual cues with complex features extracted by the deep learning models.

#### 2.4.2 Deep learning‑based Models Considered

Five widely used pre-trained deep neural network architectures were selected and trained for classifying fungal images:

VGG16, a prominent convolutional neural network architecture, emerged in 2014 as an extension of the Visual Geometry Group's (VGG) research efforts [34]. The architecture of VGG16 played a crucial role in demonstrating the significance of deep networks for image classification tasks. Its straightforward design and reliance on smaller convolutional filters showcased the potential benefits of increased model depth. Despite its relatively high computational demands due to a substantial number of parameters, VGG16 became a benchmark in the field, influencing subsequent developments in convolutional neural network design.

ResNet50 is another CNN used in the model training. ResNet50 is a renowned convolutional neural network model introduced in 2015 [35]. Part of the ResNet (Residual Network) family, ResNet50 specifically consists of 50 layers. This architecture addresses the degradation problem in deep networks, a phenomenon was increasing network depth results in diminishing accuracy. The concept of residual learning introduced by ResNet has had a lasting impact on subsequent architectures, influencing the development of deep learning models across various domains [36].

MobileNetV2 is another tested deep architecture was proposed as an evolution of the MobileNet architecture. The primary goal was to enhance the efficiency and performance of lightweight models suitable for deployment on mobile and edge devices [37]. One notable aspect of MobileNetV2 is its use of a "linear bottleneck" design, which involves a combination of depth wise separable convolutions and linear projections. This design choice enables better utilization of model capacity, leading to improved accuracy and efficiency.

DenseNet121 was proposed as an evolution of traditional convolutional neural networks with the aim of addressing issues related to feature reuse, vanishing gradients, and improving overall model efficiency [38]. DenseNet121 is one of the variants of the DenseNet architecture, with different numbers representing different depths of the network. The densely connected structure of DenseNet121 allows for improved feature propagation, mitigating the vanishing gradient problem and promoting better information flow through the network. DenseNet models have demonstrated strong performance in image classification tasks and are known for their parameter efficiency and accuracy, especially when the dataset is not extensive [39].

ViT, or Vision Transformer, represents a groundbreaking approach to computer vision by utilizing transformer architectures, originally designed for natural language processing, for image classification tasks [40], [41]. Before ViT, Convolutional Neural Networks (CNNs) dominated image classification tasks. However, ViT introduced a paradigm shift by demonstrating the effectiveness of transformers in processing image data directly without relying on predefined grid-like structures such as those in CNNs. ViT breaks down an input image into fixed-size patches, and each patch is linearly embedded into a flat vector. These vectors are then treated as sequences of tokens, similar to how words are treated in natural language processing tasks. The transformer architecture is applied to these tokenized image patches, allowing for capturing long-range dependencies and relationships between different parts of the image. Key components of ViT include the self-attention mechanism, which enables the model to focus on different parts of the input image when making predictions. ViT has demonstrated remarkable performance on various image classification benchmarks and has the advantage of being highly scalable. It can be pre-trained on large datasets and fine-tuned for specific tasks [42].

#### 2.4.3 Adam Optimizer

The deep learning models were trained using the Adam optimizer, a popular choice for deep learning tasks, with an initial learning rate set to 0.001. The use of the Adam optimizer facilitates efficient optimization of the model's weights during the training process [43]. Additionally, a batch.

size of 16 was employed, indicating that the model was updated based on 16 samples at a time during each iteration. This batch size strikes a balance between computational efficiency and model convergence, allowing the model to generalize well to the diverse fungal strains present in the training dataset. These hyperparameter choices contribute to the robustness and adaptability of the developed model, ensuring its effectiveness in classifying pathogen-fungal interactions.

#### 2.4.4 Software and Hardware

The code utilized in this study was implemented in Python and TensorFlow library version 2.15. The process of training the deep learning models was performed on Google Colab pro plus, using NVIDIA GPU A100–40G. The source code for this study is available on GitHub at the provided link (https://github.com/BDD-G/fungal_interaction/).

## 3 Results

We evaluated the performance of each deep learning model through 5-fold cross validation in order to assure that the obtained results are reliable and fair. To assess the performance of the models, four metrics have been employed for each algorithm: precision (P), recall (R), F1-score, and accuracy (Acc). Precision (P) serves as a metric evaluating the accuracy of a classification model. In more straightforward terms, precision gauges how accurately the model identifies instances predicted as positive, determining the actual positivity in Eq. 1.

(1) $$\;\mathrm{Precision}=\frac{\mathrm{TruePositive}}{\mathrm{TruePositive}+\mathrm{FalsePositive}}$$

Another critical metric in the evaluation of classification models is Recall (R). It measures the model's capacity to capture and correctly identify all pertinent instances in the dataset, as illustrated in Eq. 2.

(2) $$\;\mathrm{Recall}=\frac{\mathrm{True Positive}}{\mathrm{True Positive}+\mathrm{False Negative}}$$

The F1-Score is calculated as the harmonic mean of precision and recall. This mathematical relationship is explicitly conveyed in Eq. 3.

(3) $$\;F1=\frac{2*\mathrm{Precision}*\mathrm{Recall}}{\mathrm{Precision}+\mathrm{Recall}}$$

The accuracy score (Acc) is computed for all trained models, representing the ratio of correct predictions to the total number of samples. This calculation is presented in Eq. 4.

(4) $$\;\mathrm{Accuracy}\left(\mathrm{Acc}\right)=\frac{\mathrm{True Positive}+\mathrm{True Negative}}{\mathrm{True Positive}+\mathrm{FalsePositive}+\mathrm{TrueNegative}+\mathrm{false Negative}}$$

**Fig. 5:**
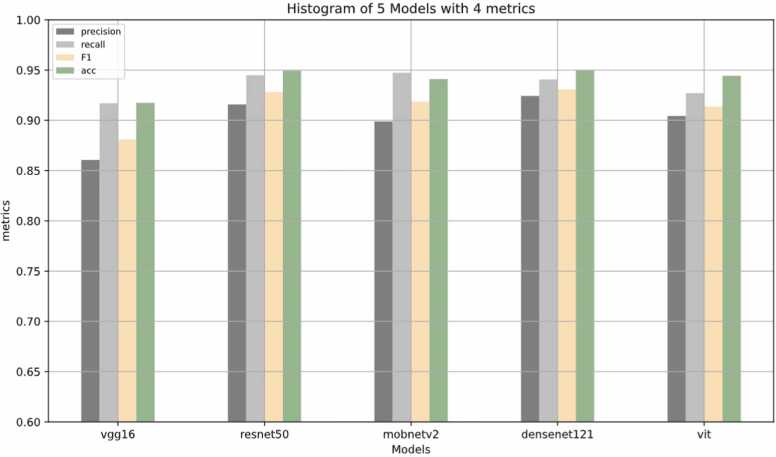
Histogram plots of four metrics for the model. The histogram is based on the average values of 5 folds. In other words, this histogram demonstrates the last column of Table 1.

**Fig. 6:**
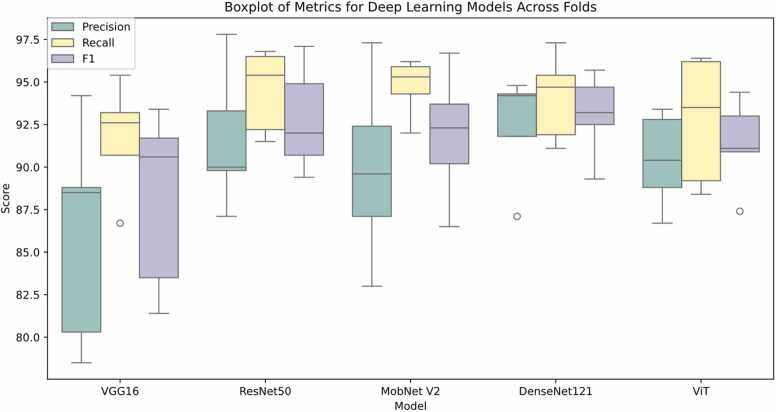
Boxplot of the performance of deep learning models across five folds.

The results of various pre-trained models, including Precision, Recall, and F1-score, are presented in Table 1. Fig. 5 displays histogram plots illustrating the metrics for the five models. These metrics were assessed as an average of over five iterations (corresponding to 5-fold cross-validation) conducted in each run during the training process. Additionally, Fig. 6 presents boxplots of the metrics across five folds for all models.

**Table 1:** The results of different pre-trained models. Precision, Recall, and F1-score presented in this table are macro average of the three classes.

Models	Fold 1			Fold 2			Fold 3			Fold 4			Fold 5			Average			
	P	R	F1	P	R	F1	P	R	F1	P	R	F1	P	R	F1	P	R	F1	Acc
VGG16	78.5	86.7	81.4	80.3	90.7	83.5	88.5	93.2	90.6	88.8	95.4	91.7	94.2	92.6	93.4	86.1	91.7	88.1	91.7
ResNet50	87.1	92.2	89.4	89.8	95.4	92	90	91.5	90.7	93.3	96.8	94.9	97.8	96.5	97.1	91.6	94.5	92.8	94.9
MobNet V2	83.0	92.0	86.5	87.1	94.3	90.2	89.6	95.9	92.3	92.4	95.3	93.7	97.3	96.2	96.7	89.9	94.7	91.9	94.1
DenseNet121	87.1	91.9	89.3	91.8	95.4	93.2	94.8	94.7	94.7	94.3	97.3	95.7	94.2	91.1	92.5	92.4	94.1	93.1	95.0
ViT	88.8	93.5	90.9	86.7	88.4	87.4	90.4	96.4	93.0	92.8	96.2	94.4	93.4	89.2	91.1	90.4	92.7	91.4	94.5

**Fig. 7:**
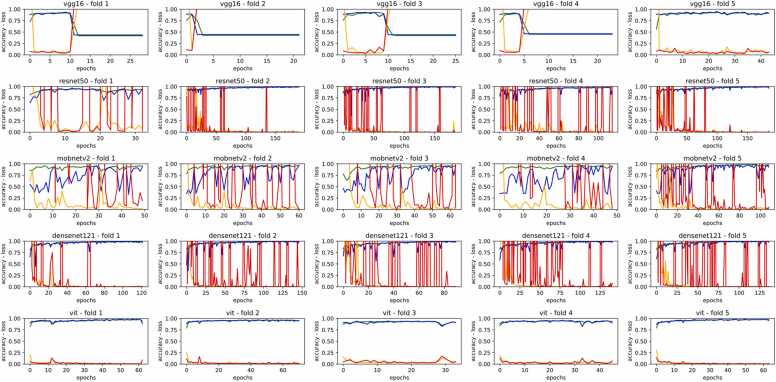
Plots of the training process for the five models for each fold. All models were supposed to be trained with 200 epochs, however, Early Stopping flag with 20 epochs monitoring (or patience in TensorFlow library) was activated to prevent the models from overfitting.

Fig. 7 illustrates the plots depicting the accuracy and loss of training data as well as validation data for five pre-trained models. The maximum value of epochs for training the models was 200, however, we activated Early Stopping flag with 20 epochs monitoring (patience in TensorFlow library) to prevent the models from overfitting. Therefore, if a model did not show improvement in loss values for validation data after 20 epochs, the training process would be ceased, and the best weights would be retained as the parameters of the trained model. As depicted in Fig. 7, the VGG16 model achieves satisfactory training within the first two epochs across all five folds, but it quickly begins to overfit. In contrast, ResNet50 exhibits a generally steady reduction in the loss values for both training and validation, despite experiencing occasional fluctuations. MobileNet-V2, on the other hand, displays significant volatility throughout its training, indicating a lack of robustness. DenseNet121 shows a steady decrease in loss values, akin to ResNet50, but with some fluctuations. However, DenseNet121 ends with lower loss values and accuracy compared to ResNet50. Meanwhile, the ViT model, which is based on transformers, maintains remarkable stability in its training across all folds, challenging the performance consistency of the other models. This stability can be attributed to the architectural design of the ViT, which relies on transformer blocks.

The initial learning rate and batch size were set at 0.001 and 16, respectively. The metrics were evaluated as an average over the five iterations (for 5-fold cross validation) performed in each run during training. Also, it is worth mentioning that all models were trained with the trained weights of ImageNet database. The evaluation of various pre-trained models for the classification yields promising results across multiple metrics.

The VGG16 model demonstrates commendable accuracy, precision, recall, and F1-Score, showcasing its efficacy in correctly identifying positive instances. Notably, VGG16 achieves a high average precision of 91.7 %, indicating its ability to minimize false positives. ResNet50 and ViT exhibit consistent and superior performance, particularly evident in their high precision and recall values across all folds. ResNet50, with an average accuracy of 94.9 %, stands out as a robust choice for fungal image classification. Moreover, ViT showcases a balance between precision and recall, underlining its competence in capturing relevant instances within the dataset. DenseNet121 also proves to be a reliable model, excelling in precision and maintaining a high accuracy of 95.0 %. The overall consistency of results across different folds and the strong average performance affirms the suitability of these pre-trained models for accurate fungal image classification, emphasizing their potential in real-world applications.

## 4 Discussion and conclusion

Machine learning algorithms have proven crucial in automating classification in diverse biological systems, and our study addresses a critical gap in existing automated systems [44]. While prior tools focused on bacterial and yeast species [45], our work introduces machine learning for the classification of fungal-fungal interactions, marking a noteworthy advancement, especially given the historical lack of fully automated evaluation tools for classical competition assays.

Leveraging computer vision and machine learning techniques, our approach excels in classifying fungal challenges, demonstrating its effectiveness in fully automated fungal-fungal interaction assays. The success of our developed model in automating classification is attributed to the size and quality of the training data, which includes a diverse collection of isolates from the IBT strain collection. This extensive dataset allows the model to learn intricate patterns associated with fungal challenges, contributing to its robustness. Emphasizing the importance of data quality, standardized images of 96-well microtiter plates provide consistent and reliable inputs, ensuring the model's generalizability across various experimental setups. However, the model’s performance may be affected by variations in image quality, such as fluctuations in lighting conditions or resolution. While we employed standardized image capture techniques, future work could explore preprocessing methods to further minimize the impact of image inconsistencies. Enhancing image quality prior to classification may lead to more reliable results, particularly when working with varied experimental setups.

One limitation is its inability to fully differentiate interactions based on growth rate alone. For instance, a strain that simply grows faster than its competitor might appear inhibitory, though the interaction may not be truly antagonistic. To address this, we incorporated control wells in which each fungus was grown individually, allowing us to compare growth rates and surface area coverage in co-culture versus single-culture conditions. In ambiguous cases, further assessments can be conducted by transferring interactions to larger formats, such as 9 cm Petri dishes, to allow for detailed analysis of growth dynamics over time. This approach enables a clearer distinction between genuine antagonistic interactions and competitive growth advantages.

While this study focuses on *Fusarium graminearum,* a highly pigmented species, a potential limitation of the technique lies in its reliance on pigmentation to distinguish outcomes. For fungi with less distinctive pigmentation, additional morphological features—such as colony structure and growth patterns—may serve as reliable indicators for classification. Future studies could explore specific model adaptations to expand classification capabilities across a broader range of fungal species.

In cases where visual differentiation between competing organisms was challenging, such as wells where both fungi exhibited similar growth patterns or where there appeared to be a “tie” (e.g., F9, C12, D9 in Fig. 1), classification relied on a combination of expert judgment and observed growth patterns. Visual indicators, such as the degree of growth inhibition or dominance over time, provided essential context that simple quantification of red area or color alone could not achieve reliably. While quantifying the red area is straightforward, it lacks the sensitivity to capture nuanced interaction patterns, making expert interpretation crucial in these ambiguous cases. Although our study primarily focused on the automated classification of fungal-fungal interactions, we observed that certain strains demonstrated more consistent antagonism against *Fusarium graminearum* than others. While no specific phylogenetic patterns were identified, these preliminary observations suggest that certain biological traits may influence competitive success. Further research into these interactions could reveal underlying mechanisms, whether chemical or structural, that contribute to biocontrol efficacy.

The model's outstanding precision, exemplified by high values across various folds, underscores its efficacy in accurately classifying fungal-fungal interactions, crucial for reliability. The performance analysis reveals that DenseNet121 consistently achieved the highest precision (92.4), recall (94.1), F1-score (93.1), and accuracy (95.0) among the models. This demonstrates the robustness and superiority of DenseNet121 in capturing the complexities of the dataset and making precise predictions. The results affirm its potential as a valuable tool in the context of fungal-fungal interaction studies. While the model demonstrates high accuracy, it does not reach the 100 % threshold. This limitation bears consequences for the screening process, introducing the possibility of overlooking some positive hits. Nevertheless, it operates efficiently as an initial screening step. To bolster the screening process's reliability and minimize the risk of missing valuable findings, we incorporate multiple replicates of fungal-fungal interactions. Another concern is the model's sensitivity to fungal strains not represented in the training dataset. Since the dataset comprises only known fungal isolates, the model may not generalize effectively to novel or rare strains. Addressing this limitation will require additional training with a more diverse set of fungal species or the application of data augmentation techniques to enhance the model's adaptability to new strains.

Through 5-fold cross-validation results, the model showcases robust performance, highlighting its adaptability to various scenarios. While the model exhibits high accuracy, it still has limitations that bear implications for the screening process, as there's a chance of missing some positive hits. However, it functions effectively as an initial screening step. To enhance the reliability of the screening process and minimize the risk of overlooking valuable findings, we incorporate multiple replicates of fungal-fungal interactions.

Beyond automated classification, our approach opens avenues for further biological analysis of the identified biocontrol strains. Once a candidate strain is selected, metabolites or proteins produced by the biocontrol strains could be analyzed using mass spectrometry or other bioanalytical techniques to characterize the active compounds responsible for inhibiting *Fusarium* growth. Such biochemical insights can guide the development of biocontrol agents by correlating phenotypic interactions with specific biochemical properties. Such a workflow enables researchers to not only identify potential biocontrol strains but also to understand the mechanisms behind their antifungal activities. A natural next step would involve validating these findings in larger cultivation space such as 9 cm petri dishes and assays that more closely simulate natural environments, such as whole-plant assays and, eventually, field trials. Additionally, investigating the mode of action would be valuable for understanding the selectivity of biocontrol organisms and refining application strategies to enhance both efficacy and environmental compatibility.

We focused primarily on *Fusarium graminearum* in this study due to its clear pigmentation and the availability of well-characterized data. Future studies could explore applying this model to other pathogens with different pigmentation and growth characteristics.

The development of our approach has important implications for biotechnological applications, particularly in biological control for managing crop diseases. By automating the classification process, the model addresses the limitations of manual inspection, reducing time consumption and introducing standardization. The model's high precision and scalability align with the urgency to identify efficient biological solutions for disease management, ensuring food security and minimizing the environmental footprint of food production.

An additional advantage is that it reduces the needed manpower for analysis and introduces an objective classifying of the plates in microbe-microbe interaction studies. This not only streamlines the analytical process but also enhances the reliability and reproducibility of the results, making it a valuable tool for advancing research in the field of fungal-fungal interactions and agricultural disease control.

In conclusion, our research presents an automated classification of fungal-fungal interactions, showcasing commendable precision and scalability. The integration of artificial intelligence, specifically computer vision and machine learning, into the field of mycology signifies a transformative shift in the evaluation of fungal interactions. This study uses convolutional neural networks and deep learning for image analysis, highlighting its modern approach.

The utilization of this model in mycological studies offers a promising avenue for the efficient identification of biocontrol organisms, contributing significantly to sustainable agriculture and disease management. The models consistently classify fungal strains with comparable accuracy across various architectures, eliminating subjectivity between annotators. This standardization and reproducibility fostered by the model provides a more robust and reliable approach to fungal interaction analysis, advancing the field of mycology and establishing a foundation for future research.

## Funding

This research was funded by the 10.13039/100012774Innovation Fund Denmark under the 'Grand Solutions' funding instrument (grant number 0224–00092B) for the 'Smarter AgroBiological Screening' (SABS) project.

## CRediT authorship contribution statement

**Marjan Mansourvar:** Writing – original draft, Methodology, Formal analysis, Software, Validation, Visualization, Supervision, Funding acquisition. **Jonathan Funk:** Writing - original draft, Methodology, Formal analysis, Software, Validation, Visualization. **Søren Dalsgård Petersen:** Review & editing, Data curation. **Sajad Tavakoli:** Formal analysis, Software, Methodology, Validation, Visualization. **Jakob Blæsbjerg Hoof:** Supervision, Resources, Review & editing. **David Llorente Corcoles:** Validation. **Sabrina M. Pittroff:** Data acquisition. **Lars Jelsbak:** Resources, Funding acquisition. **Niels Bjerg Jensen:** Project administration. **Ling Ding:** Resources, review & editing, Funding acquisition. **Rasmus John Normand Frandsen:** Writing – review & editing, Supervision, Funding acquisition.
